# Hospital Acquired Pathogenic *Escherichia coli* from Clinical and Hospital Water Samples of Quetta Balochistan

**DOI:** 10.1155/2022/6495044

**Published:** 2022-10-13

**Authors:** Ali Akbar, Wajeeha Naeem, Faiza Liaqat, Muhammad Bilal Sadiq, Muhammad Shafee, Zareen Gul, Shabir Ahmad Khan, Hasina Mengal, Su Hlaing Chein, Sadia Qasim, Muhammad Arshad, Fazal Ur Rehman, Hassan Sher

**Affiliations:** ^1^Department of Microbiology, University of Balochistan Quetta, Quetta 87300, Pakistan; ^2^School of Life Sciences, Forman Christian College (A Chartered University), Lahore, Pakistan; ^3^CASVAB University of Balochistan Quetta, Quetta 87300, Pakistan; ^4^Department of Botany, University of Balochistan Quetta, Quetta 87300, Pakistan; ^5^Spectrum Sustainable Development Knowledge Network, Yangon, Myanmar; ^6^The University of Child Health Sciences and Children's Hospital Lahore, Lahore, Pakistan; ^7^Department of Basic Science, Jhang-Campus University of Veterinary & Animal Sciences, Lahore, Pakistan; ^8^Centre for Plant Sciences and Biodiversity, University of Swat, Charbagh, Swat 19120, Khyber Pakhtunkhwa, Pakistan

## Abstract

A study was conducted to determine the prevalence and drug resistance of *Escherichia coli* present in urinary tract infected patients and hospital drinking water. A total of eighty urine samples from clinically suspected patients and thirty tap water samples from hospital vicinity were collected and analyzed for the presence of *E. coli*. The isolates were preliminary identified based on morphological characteristics, biochemical test and further confirmed by polymerase chain reaction (PCR) using *uidA* primer. Isolates were subjected to antibiogram studies and analyzed for the presence of drug resistance (*ESBL blaCTX-M-15*, *tetA*, and *TMP-SMX dfrA1*) and pathogenicity associated pyelonephritis-associated pili (*PAP*) and *Heat-labile (LT) toxin* genes. Urine samples 19/80 (23.75%) and water samples 8/30 (26.7%) were found contaminated with *E. coli*. It was found that 12/19 (63%) bacterial isolates were extended spectrum beta-lactamase (ESBL) producers in clinical and 6/8 (75%) in water isolates whereas tetracycline resistance in clinical and water isolates was 11/19 (58%) and 6/8 (75%), respectively. The trimethoprim resistance gene was confirmed in 12/19 (63%) in clinical and 2/8 (25%) in water isolates. All the clinical and water isolates were found carrying pili *PAP* gene. It was concluded that the presence of drug resistant and pathogenic *E. coli* in clinical and water samples is extremely alarming for public health due to cross contamination and bacterial transfer from clinical samples to water and vice versa.

## 1. Introduction

Antibiotics are essential tool against pathogenic bacterial infections but the rapid emergence of antibiotic resistant pathogens is currently creating an alarming situation [1]. Drug resistance is a global public health concern, associated with more than 700,000 deaths annually [2]. This growing threat necessitates the action to prevent the emergence of new resistant bacterial strains and the spread of existing ones in the environment from humans and animals [3]. Antibiotic resistant bacteria have been found in wastewater, surface water, and drinking water. Water is regarded to be the most important medium for the transmission of antibiotic resistance. This is especially true in low-income nations, where lack of sanitation and hygiene practices aggravates the problem [4]. Hospital sanitation water is associated with the presence of antibiotic residues and resistant microorganisms which are delivered into the aquatic ecosystem [5]. Each year, hospitals in Pakistan are roughly generating 25,000 tons of wastes with antibiotic residues ranging from 1.4 g/L to 236.6 g/L, which is thrown into surface water untreated [6]. Due to lack of appropriate management, drinking water is regularly mixed with sewage water and contaminated hospital effluent that poses a serious threat to the human population. In such conditions, the presence of antibiotic resistant bacteria increases the possibility of horizontal genetic transmission between microbes, leading to the emergence of multidrug resistant bacteria [7, 8]. *E. coli* can easily acquire drug resistance genes and is responsible for a variety of human diseases, including traveler's and infantile diarrhea, urinary tract infections, septicemia, hemolytic uremic syndrome, newborn meningitis, and pneumonia [9, 10]. *E. coli* is a commensal, opportunistic pathogen that is found in animals' gastrointestinal tracts as well as in the environment, such as in water and soil [11]. In this work, *E. coli* bacteria isolated from clinical and water sources were evaluated for drug resistance pattern and virulence potential. The aim of this study was to determine the prevalence of pathogenic and drug resistant hospital acquired *E. coli* from clinical and hospital water samples. The findings of this study will be useful globally in antibiotic treatment and monitoring the resistance patterns over time in developing and underdeveloped regions.

## 2. Methodology

### 2.1. Sampling

Urine samples (*n* = 80) were collected aseptically in sterile sampling containers from the patients having clinical signs such as pelvic pain, burning sensation during urination, high fever, and blood in urine. All the samples were collected using convenience random sampling technique from Bolan Medical Complex Hospital and Provincial Sandeman Hospital Quetta, city of Pakistan from April to December 2018, following all the safety procedures and protocols. Informed consent was obtained from all the participants.

Drinking tap water samples (*n* = 30) were collected from hospital vicinity aseptically using sterilized glass bottles. All the samples (clinical and water) were transported to Center for Advanced Studies in Vaccinology and Bacteriology, University of Balochistan, and during transportation, temperature (0–4°C) was maintained. The samples were immediately (within 6 h of collection) processed for the isolation of target pathogen *E. coli*.

### 2.2. Isolation and Identification

The clinical samples were streak directly over the surface of sterile MacConkey agar (MCA) (Oxoid, UK) plates, while the water samples were first enriched in lactose broth for 6–16 h at 37°C, then transferred to MCA, and incubated at 37°C for 24 h [12].

Suspected lactose fermenting pink colonies from MCA were transferred to Eosin Methylene Blue (EMB) agar (Oxoid, UK) and incubated at 37°C for 16–24 h [13]. Colonies with green metallic sheen and typically resembling *E. coli* were further characterized. Gram's staining, Catalase, Oxidase, Sulfide, Indole Motility, Methyl Red, Voges Proskauer, Simmon citrate utilization, and urease tests were performed for the biochemical conformation of the *E. coli* isolates [12].

### 2.3. Molecular Identification

The preliminary identified *E. coli* isolates were subjected to molecular identification using *E. coli UidA* primer that amplifies 147 bp gene size product [14]. Bacterial DNA was extracted by using the DNA Mini Kit (Qiagen), and polymerase chain reaction (PCR) was carried out by the use of specific oligonucleotide primers. PCR mixture (25 *μ*l) was comprised of master mix (12.5 *μ*l), molecular grade water (7.5 *μ*l), 1 *μ*l of primer (forward and reverse), and 3 *μ*l of DNA template. Using a thermocycler (Bio-Rad, USA), PCR was carried out (Table 1). PCR products were analyzed by electrophoresis in 1.2% agarose gel, and visualization of bands was done in transilluminator (Dolphin View-Wealtec, USA) under UV light.

### 2.4. Antibiogram of Isolates

Antibiogram of *E. coli* isolated was determined through Kirby-Bauer disk diffusion procedure using Mueller Hinton agar (MHA) (Oxoid, UK), following Clinical Laboratory and Standard Institute (CLSI) guidelines [15]. Overnight grown culture was assorted in 1 mL of sterile saline solution equating the turbidity to 0.5 McFarland standard followed by swabbing on MHA [16]. Twelve frequently prescribed antibiotics were impregnated on the surface of MHA comprising of tetracycline (30 *μ*g), gentamycin (10 *μ*g), chloramphenicol (30 *μ*g), trimethoprim-sulphamethoxazole (25 *μ*g), ciprofloxacin (5 *μ*g), ceftazidime (30 *μ*g), amikacin (30 *μ*g), cefixime (5 *μ*g), Imipenem (10 *μ*g), amoxicillin (30 *μ*g), amoxicillin + Clavulanic Acid (30 *μ*g), and cefotaxime (30 *μ*g). After incubation of 24 h at 37°C, diameter of inhibition zone was measured.

### 2.5. ESBL Conformation Test

The double disc synergy test was done on MHA to determine extended spectrum beta-lactamase (ESBL) producing isolates. Ceftazidime and Cefotaxime discs were spaced 15 mm away from an amoxicillin-clavulanic acid (20/10 *μ*g) disc in the plate's middle [17].

### 2.6. Molecular Detection of Drug Resistance Genes

Genes responsible for drug resistances against selected antibiotics were determined using gene specific primers for the targeted genes including *ESBL blaCTX-M-15* gene that amplifies 996 bp gene size product [13], tetracycline *tetB* gene that amplifies 634 bp gene size product [18], and *TMP-SMX dfrA1* resistance gene that amplifies 367 bp gene size product [19].

By using thermocycler, PCR mixture (25 *μ*l) was used to amplify *ESBL blaCTX-M-15* gene, *tetB* gene, and *TMP-SMX dfrA1* (Table 1).

### 2.7. Molecular Detection of Pathogenicity-Related Genes

Two virulence genes *PAP* [20] and *Heat-labile toxin* [21] were targeted to determine the pathogenicity potential of the isolates by PCR. By using thermocycler, PCR mixture (25 *μ*l) was used to amplify *PAP* gene (initial denaturing 3 min at 94°C, 35 cycles of 1 min at 94°C, 1 min at 58°C, 1 min at 72°C followed by final extension of 5 min at 72°C) and *heat-labile toxin* gene (initial denaturing 3 min at 94°C, 35 cycles of 1 min at 94°C, 1 min at 58°C, and 1 min at 72°C and final extension for 5 min at 72°C).

### 2.8. Statistical Analysis

Statistical calculation of the data was done by using Microsoft Excel. Chi-square goodness-of-fit test was used for the determination of difference in resistance. *p* values < 0.05 were considered statistically significant.

## 3. Results and Discussion

**Figure 1:**
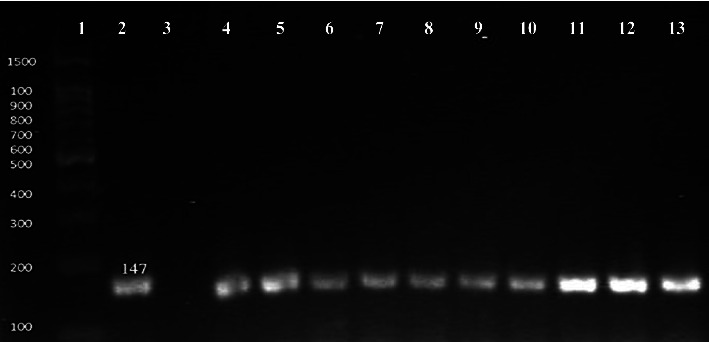
Molecular conformation of *E. coli* by *uidA* gene. Number 1: 100 bp DNA ladder; number 2: positive control; number 3: negative control; number 4 to 13: positive samples.

A total 110 sample comprising 80 urine and 30 tap water samples were analyzed for the detection of *E. coli.* Out of total 110 samples, 25% (27/110) were found contaminated with *E. coli*, whereas 23.75% (19/80) urine sample was positive for the presence of the target pathogen, while 26.7% (8/30) water sample was confirmed with the presence of *E. coli* through conventional and molecular methods (PCR) targeting *uidA* gene (Figure 1).

Due to frequent and unnecessary use of antibiotics, *E. coli* is becoming drug resistant, making it a superbug and a huge public health risk. Antibiotic susceptibility of *E. coli* isolates must be determined for the proper and precise prescriptions. If proper management and precautions have not been taken for the prevention of drug resistance, the current death toll 700,000 per year can be increased to ten million mortality rate every year throughout the globe by year 2050 [22]. Drug resistance is a barrier to universal health care and achieving sustainable development goals in the areas of food security, clean water, sanitation, and health [23].

Contrary to our finding, Talukdar et al. [13] reported 63% prevalence of *E. coli,* in household water, which was relatively high compared to our findings. Similar to our study, Shimpoh et al. [24] used *uidA* primer to identify *E. coli* strains from clinical samples collected from Japanese patients and reported a high detection rate of 51%.

### 3.1. Antibiogram

**Figure 2:**
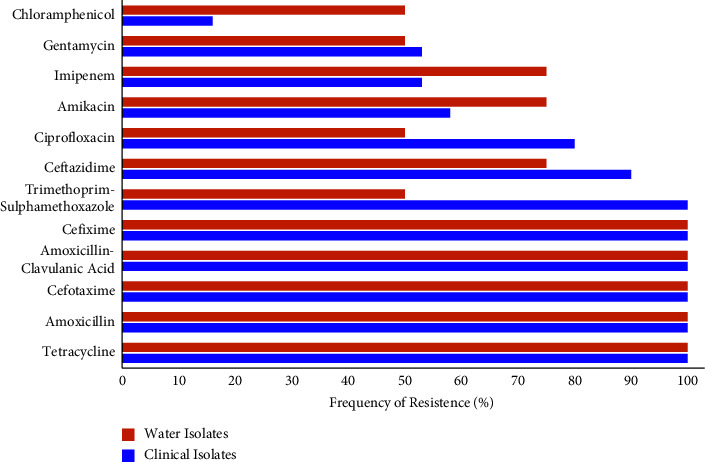
Drug resistance pattern in *E. coli* isolates from clinical and water samples.

Twelve commercially available antibiotic discs were used to determine the antibiogram of the isolates. A majority of the isolates showed resistance to one or more antibiotics. Antimicrobial susceptibility test results revealed that few isolates showed susceptibility against chloramphenicol, amikacin, and imipenem while large number of *E. coli* isolates showed 100% (27/27) resistance to tetracycline, amoxicillin, amoxicillin-clavulanic acid, cefotaxime, and cefixime in clinical and water isolates (Figure 2).

In agreement with our results, previous reports (23, 24) documented that *E. coli* showed resistance against tetracycline and ceftazidime. Comparing with our work, research conducted in Bangladesh revealed that *E. coli* showed higher resistance against tetracycline (91.22%), trimethoprim (29.82%), and chloramphenicol 29% [25]. Another study demonstrated that *E. coli* was resistant to ampicillin (98%), tetracycline (23%), and trimethoprim-sulpha methoxazole (38%), respectively [26]. Contrary to our findings, previously a lower resistance in *E. coli* isolates, 45% tetracycline, 36% trimethoprim-sulfamthoxazole, 17% ciprofloxacin, 8% chloramphenicol, and 1% gentamycin was documented [13]. Scaria et al. [27] studied that all *E. coli* strains were found resistant to streptomycin (95%), tetracycline, ampicillin (47%), and kanamycin (91%).

Moges et al. [28] reported 100% ampicillin resistant and 38% tetracycline resistant *E. coli* isolated from hospital wastewater. They also reported high resistance of *E. coli* isolated from hospital wastewater to cefotaxime (38%), trimethoprim/sulfamethoxazole (38%), and cephalothin (23%). Tetracycline (21.45%), trimethoprim/sulfamethoxazole (18.56%), ampicillin (11.32%), ciprofloxacin (8.25%), amikacin (7.22%), gentamicin (5.15%), and cefotaxime (4.12%) resistance was found in *E. coli* isolates from contaminated water in Ghana [29]. These investigations have demonstrated that the habitat has a significant impact on *E. coli* antibiotic resistance due to exposure to various levels of drug residues in surrounding environment.

Out of 27 isolates, only 5 were susceptible to ceftazidime while other isolates showed resistance against various commercially available antibiotics. A research study conducted in Saudi Arabia reported that ESBL producing *E. coli* isolate demonstrated 100% resistance against amoxicillin/clavulanic acid and showed higher resistance rate against ceftazidime 100% and amikacin 54% comparing with present results 80% and 50%, respectively [30]. The current study suggested that the commercially available antibiotics such as amikacin, ceftazidime, and imipenem are potent against ESBL *E. coli* isolates.

### 3.2. Drug Resistance and Virulence Gene

**Figure 3:**
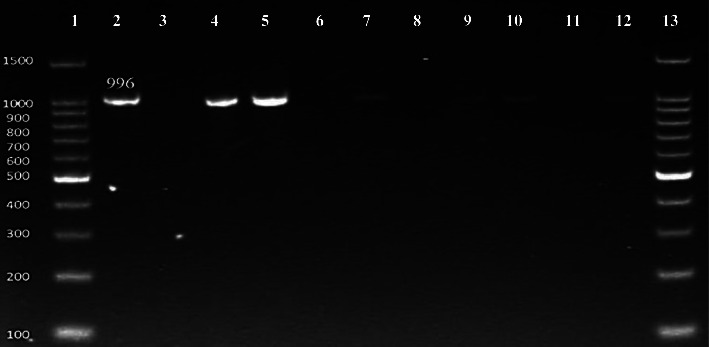
Molecular detection of ESBLs (*blaCTX-M*-15) gene (996 bp). Number 1 and 13: 100 bp DNA ladder; number 2: positive control; number 3, 6, 8, 11: negative samples; number 4, 5, 7, 9, 10, 12: positive samples.

**Figure 4:**
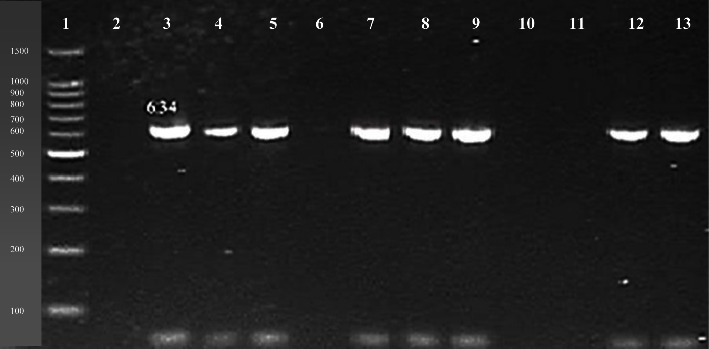
Molecular detection of *tetB* drug resistance gene (634 bp). Number 1: 100 bp DNA ladder; number 2, 6, 10, 11: negative samples; number 3: positive control; number 4, 5, 7, 8, 9, 12, 13: positive samples.

**Figure 5:**
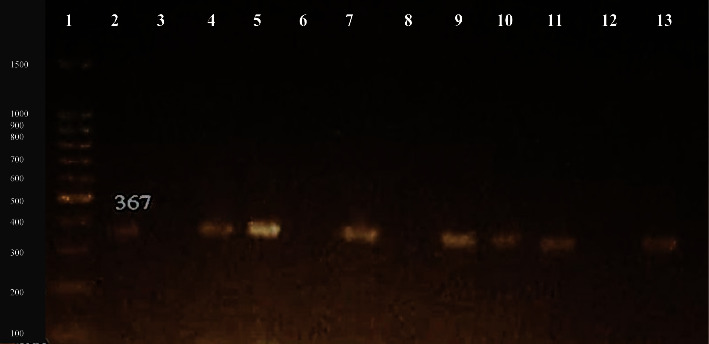
Molecular detection of *dfrA1* gene (367 bp). Number 1: 100 bp DNA ladder; number 2: positive control; number 3, 6, 8, 12: negative samples; number 4, 5, 7, 9, 10, 11, 13: positive samples.

The results for the identification of drug resistance genes revealed that *ESBL blaCTX-M-15* gene was found in 63% (12/19) of clinical and 75% (6/8) of tap water isolates (Figure 3) followed by tetracycline resistance gene *tetA* 58% (11/19) in clinical and 75% (6/8) in tap water isolates (Figure 4). The *TMP-SMX dfrA1* resistance gene was found in 63% (12/19) of clinical and 25% (2/8) of tap water isolates, respectively (Figure 5).

In comparison to our findings, a high prevalence rate (66%) of *bla CTX-M-15 ESBL* gene was found in *E. coli* isolate from water samples [31]. Comparing with present work, a research work reported lower prevalence rate of *CTX-M-15 ESBL* gene (43%) in *E. coli* clinical isolates [13]. Lyimo et al. [32] studied the prevalence of *blacCTX-M-19* and *blaCTX-M-15* in *E. coli* from water samples and reported lower prevalence comparing with the current study. In a study conducted in Pakistan, blaCTX-M gene was found in 29% *E. coli* isolates from water sample. *BlaSHV* and *blaTEM* were shown to impart resistance to several antibiotics in their investigation, and they were the most widespread in *E. coli*, which contained the highest number of resistance genes, including *blaCTXM, blaOXA, and blaNDM*. [33].

Tetracycline (*tetA*, *tetB*, and *tetC*) and trimethoprim-sulfamethoxazole resistance genes were investigated by Ibekwe et al. [34] in *E. coli*. The results of their study revealed that 10 *E. coli* isolates were positive for *tetB* gene and 10 isolates were also positive for *dfrA1* gene. Comparing with the present study, a research study conducted in Iran, reported that *tetB* (53.63%) and *dfrA1* genes (36.84%) were present in PCR-confirmed *E. coli* isolates [25]. Another study documented that *tet B* and *TMP-SMX dfrA1* gene were highly prevalent in *E. coli* from clinical samples [35].

**Figure 6:**
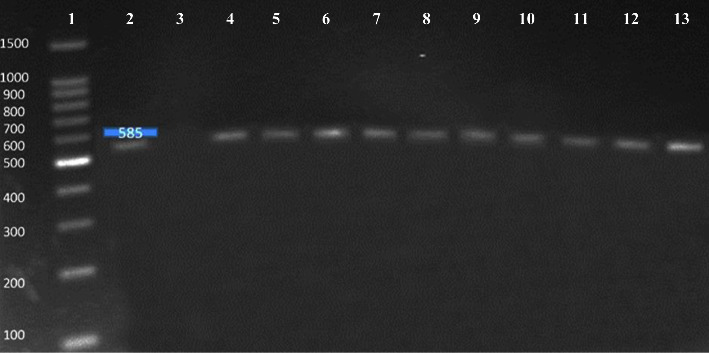
Molecular detection of *PAP* virulence gene (585 bp). Number 1: 100 bp DNA ladder; number 2: positive control; number 3: negative control; number 4 to 13: positive samples.

The virulence gene *PAP* was found in all (100%) the confirmed *E. coli* isolates (Figure 6) while *Heat-labile toxin* gene was found absent in all isolates. In a similar study, it was reported that *E. coli* isolates tested for virulence gene *PAP* and *Heat-labile toxin* (LT) revealed 18% presence of PAP gene [36] and 12.7% *LT* gene detection in the isolates [37]. In another study conducted in South Africa by Nontongana et al. [26], 2% *PAP* gene and 47% *LT* gene presence were observed in the *E. coli* isolates. In comparison to the current study, literature variations in the prevalence of *E. coli* in clinical samples and antibacterial resistance might be due to different geographical regions, difference in the level of antibiotic exposure, and variation in *E. coli* strains. A detailed study by including larger sample size and collection of samples from different sampling sites around the region may provide more comprehensive findings.

## 4. Conclusion

It was concluded in this study that *E. coli* bacteria isolated form clinical and hospital vicinity water were resistant (100%) to Tetracycline, trimethoprim-sulfamethoxazole, Cefizime, and amoxicillin. Furthermore, drug resistance (*ESBL blaCTX-M-15*, *tetA*, and *TMP-SMX dfrA1*) and pathogenicity associated *PAP* genes were also present in the bacterial isolates, which presented a serious health concern. Precautions are required to reduce the incidence of multidrug resistant *E. coli* strains among humans. In order to achieve this, strict quality control procedures should also be used to ensure that water and wastewater are properly treated.

## Figures and Tables

**Table 1:** Details of primers and their respective polymerase chain reaction conditions.

Gene	Size	Polymerase chain reaction protocol	Sequence of gene
*UidA*	147 bp	Initial denaturation was carried out for 7 min at 95°C followed by total 35 cycles of 30 sec each at 95°C; annealing for 30 sec at 55°C; extension for 30 sec at 72°C, followed by final extension at 72°C, for 7 min	F-TGGTAATTACCGAC GAAAACGGC R-ACGCGTG GTTACAGTCTTGCG

*blaCTX-M-15*	996 bp	Initial denaturation for 4 min at 96°C followed by total 34 cycles of 20 sec each at 95°C; annealing for 20 sec at 56°C; 60 sec at 72°C, followed by final extension at 72°C, for 5 min	F-CACACGTGGAATTTAGGGACT R-GCCGTCTAAGGCGATAAACA

*tetB*	634 bp	Initial denaturation for 3 min at 95°C followed by total 35 cycles of 1 min each at 94°C; annealing for 90 sec at 56°C; 60 sec at 72°C, followed by final extension at 72°C, for 8 min	F-CCTCAGCTTCTCAACGCGTG R-GCACCTTGCTGATGACTCTT

*TMP-SMX dfrA1*	367 bp	Initial denaturation for 3 min at 95°C followed by total 35 cycles of 1 min each at 94°C; annealing for 90 sec at 45°C; 60 sec at 72°C, followed by final extension at 72°C, for 8 min	F-GGAGTGCCAAAGGTGAACAGC R-GAGGCGAAGTCTTGGGTAAAAAC

*PAP*		Initial denaturing 3 min at 94°C, 35 cycles of 1 min at 94°C, 1 min at 58°C, 1 min at 72°C followed by final extension of 5 min at 72°C	F-AACCTGGCTTACGCAACTGTACCC GT R-CTG CAA AAT CAT GGA T

*Heat-labile toxin*		Initial denaturing 3 min at 94°C, 35 cycles of 1 min at 94°C, 1 min at 58°C, and 1 min at 72°C and final extension for 5 min at 72°C	F-GCACACGGAGCTCCTCAGTC R-TCCTTCATCCTT TCA ATG GCT TT

## Data Availability

Major part of the data is presented in the manuscript, and the remaining data will be made available on reasonable request.
